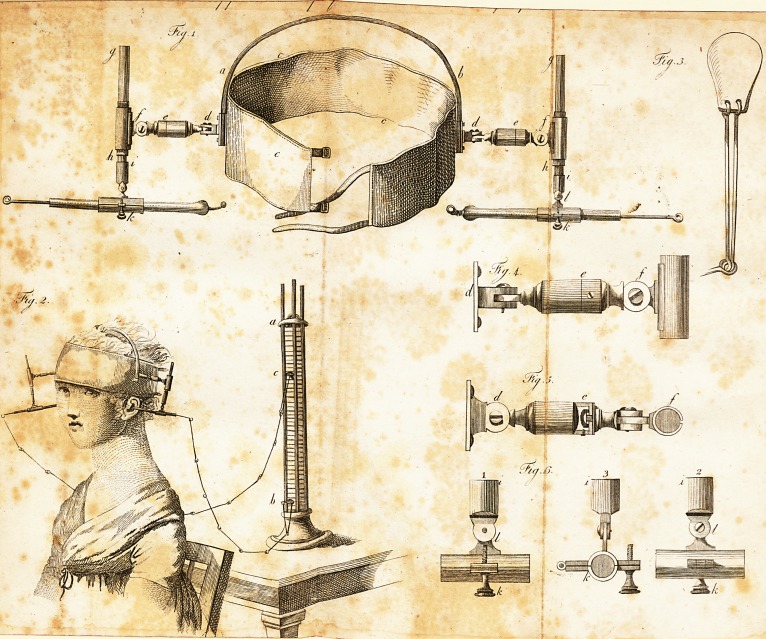# Observations and Experiments, Made with the View of Employing Galvanism for the Cure of Several Diseases

**Published:** 1802-10-01

**Authors:** 

**Affiliations:** Berlin


### *Observations and Experiments, made with the view of employing Galvanism for the Cure of several Diseases; by Dr.* GRAPENGIESSER, *of Berlin; with an Engraving.*

[ Continued from pp. 250—259. ]

Sec. 3. ON *the Action of the Galvanic Battery in general, and especially with respect to its Relation to Electricity.*—In order to analyse the properties and nature of Signor *Volta*'s remarkable apparatus, but particularly to investigate its effects on organic bodies, I made a series of experiments on different animals, as well as on myself and several of my friends, the results of which soon convinced me of its great power, and intitled me to to expect greater effects from it than thofe which I had obferved from the application of fimple Galvanifm. Indeed, there feems nothing equal to it in penetrating and exciting the nervous fyftem; hence I concluded, after what I already fo fuccefsfully experienced in employing fimple Galvanifm, that it might prove of the moft extraordinary fervice in nervous diforders, chiefly in thofe originating in debility, attended with deficient irritability and activity of the fyftem. Its fpecific action on the optic nerve, in producing the appearance of lightning in the eyes, I particularly confidered as promifing great efficacy for the cure of amaurofis. The action of the Galvanic Battery feems, in fome meafure, to have more analogy to Electricity than fimple Galvanifm; and the fenfation produced by touching the two poles with two filver fpoons, appears to fome people very fimilar to the fenfation occafioned by a ftrong fpark from the Leyden phial. Others, however, think it quite different, and defcribe the fenfation as more penetrating than common electrical fhocks of equal ftrength. Mr. *Volta* himfelf, thinks the fenfation produced by his new apparatus, more refembling the fenfation caufed by touching the Gymnotus electricus and Raia torpedo, which exactly agrees with a late opinion of Mr. Humboldt, communicated in one of his letters from America, who fays, that according to his refearches, the phenomena obferved in thofe fifhes are rather of a Galvanic than of an electrical nature. It is not yet fufficiently afcertained whether there exifts a material difference between the phenomena of Galvanifm and Electricity: With regard to the medical application, however, the former appears to differ in fome effential points from the latter, which, according to my numerous experiments, I found to be the following:

1. *Galvanism seems to penetrate much easier, and, as it were, deeper into the nerves, which seem to be its best conductors; whereas electricity is more uniformly communicated throughout the whole animal body, over the surface of which it is universally spread.*

This is proved by the fubfequent arguments.

*a.* Galvanifm produces a peculiar irritation of the optic nerve and the organs of tafte; or an appearance of light in the eyes, and a change of tafte, which always enfue whenever a fimple Galvanic chain is fhut at any part of the face that is covered with a tender epidermis; or when a circuit of Galvanifm is formed by means of the pile at thofe places to which the branches of the fifth and eighth pair extend. Although an electric fpark may likewife occafion the appearance of lightning in the eye, it muft be confiderably ftrong, and either applied immediately on the eye-ball or on the neighbouring parts. On letting letting paſs a ſtrong ſpark from the Leyden phial to the frontal nerve, an appearance of light will be perceived in the dark, which, however, greatly differs from that Galvanic phenomenon.

*b.* The phenomena obſerved, by applying the Galvaniſm on ſeparated animal parts, which are not yet wholly deprived of vital power, viz. legs of frogs, &c.

*c.* Galvaniſm, or the metallic ſtimulus, may ſerve as a means of diſtinguiſhing nerves from other organs.

*d.* On applying one conductor of the Galvanic battery to the mucous membrane of the noſe, and the other to the frontal nerves, the whole net of nerves* which is ſpread on the back of the noſe and the upper jaw will be ſeen, ſo as to be able to diſtinguiſh their courſe and ramifications.

2. *The Galvanic Fluid seems to be much easier decomposed than common Electricity within organic bodies as well as without.*

*a.* The Galvanic battery exerts a great effect on the nervous ſyſtem, and produces the moſt violent ſhocks, while it hardly affects the electrometer; and in common caſes the Galvaniſm of a battery of 100 or 150 ſtrata is not indicated but by condenſation.

*b.* Galvaniſm ſeems to ſet the inflammable bodies, as ſulphur, phoſphorus, gunpowder, &c. very eaſily on fire.

*c.* It has the power of decompoſing water; all which proves its having a greater and quicker effect on organic bodies.

3. *Its action on wounds occasioned by a blister, and on the cuticular nerves and vessels, is such as is never produced by Electricity.*

4. I have as yet been able *only to act by Galvanism on single parts, and consequently on topical diseases*; whereas the whole ſyſtem may be changed and acted upon by Electricity.

5. *Galvanism is never conducted through the dry skin*; but its action is immediately perceived as ſoon as the ſkin is wetted.

Theſe are the differences by which the action of Galvaniſm is to be diſtinguiſhed from that of Electricity, as far as it may be intereſting for the practice of medicine, on which account we forbear to mention the other differences, pointed out by ſeveral Naturaliſts, as foreign to our purpoſe.

Sec. 4. *On the different Action of the simple Galvanic Chain, and of the Battery at its two Ends or Poles.* It is known that the ſimple Galvanic chain as well as the battery, act at their poles in a different manner with reſpect to intenſity and quality. From my obſervations it appears, that the zinc ſide or zinc pole produces a greater effect than the oppoſite pole at the moment the Galvanic chain is ſhut, and as long as it remains ſo. On applying a plate of ſilver and another of zinc, connected nected by a siver or gold chain, to wounds occasioned by blisters, the pain, shocks, and convulsions of the nerves, of the lightning, in case the branches of the fifth and eighth pair should be affected, are much stronger at the zinc side than at the silver side. A lymphatic humour begins to run from both wounds, which having continued for about six or eight hours, a thick eschar or gangrenous crust arises on the wound at the zinc side, while the opposite side continues to suppurate. On touching with wet fingers, the wires of the conducting plates of a moderately strong battery, the sensation is much stronger and penetrating at the zinc wire than at the silver side, where it is merely superficial, resembling a tension, as if the finger was swoln and inflamed. When the conductors of the two poles of the pile are brought into the meatus auditorius, that of the zinc pole occasions more violent shocks, and is more piercing; and the sound and tinkling of the ear which it produces, is stronger than that which is felt at the silver side, where the sensation is rather burning. In a state of diminished excitability the zinc pole alone proves active; but the silver pole not at all. On applying the conductor of the zinc side into the nose, and on taking hold of the opposite conductor with the wetted hand, a most insupportable acute pain will be occasioned, and a most violent effort to sneeze arise; whereas, when the conductor of the silver pole is brought into the nose, the pain is more dull, without any effort to sneeze. The action of the zinc pole on the eyes and tongue is likewise more intense, painful, and penetrating than that of the silver pole.

These phenomena, however, only take place the moment the Galvanic chain is shut, or when it is suffered to remain shut; for on separating it, the organ that is in connection with the silver pole, is more affected and irritated than that at the zinc pole; but this opposite impression is only momentary, and by no means capable of abolishing the effects produced by the primitive action in the interior organisation, as has been supposed by some Naturalists; because,

*a.* There always remains, after every experiment, more or less of the sensation that was primarily produced, which would never be the case if the opposite action, occasioned at the moment the chain is separated, had entirely supplanted, and as it were, neutralized the former.

*b.* The sounding and tinkling of the ears frequently remain on the zinc side when the organs of hearing have been galvanized.

*c.* There have been some changes produced in diseases, against which Galvanism was employed, which, however, would not not have taken place if the firſt effects were aboliſhed by the contrary action.

The differences which the two poles ſhow with reſpect to the quality of irritation, are not ſo eaſily to be determined, as they are with difficulty perceived by ſeveral perſons, and by ſome not at all. The experiments, therefore, intended for this purpoſe, ſhould be accurately made and undertaken early in the morning, when the excitability is not yet diminiſhed. It is particularly through the medium of the organs of ſight and taſte, that we find ſome difference in the reſpective action of the two poles. On touching the point or ſurface of the tongue with a zinc probe, and with a ſilver probe, any part of the body which is covered with a thin epidermis is deprived of it entirely, viz. palate, gums, noſe, eye, urethra, &c. and on bringing the two metals in connection with one another, a *sourish* taſte will ariſe; whereas, when the ſilver is applied to the tongue, and the zinc to the other parts, the taſte becomes *alkaline*. This phenomenon is almoſt always obſerved, and there are few people who have not perceived it. From the analogy, therefore, which the ſimple Galvaniſm ſhows in moſt points with the Galvanic battery, we may be entitled to ſuppoſe, that it likewiſe takes place in the ſame manner at the two poles of the pile, which, however, is by no means the caſe; for moſt people perceive the ſouriſh taſte at the ſilver ſide of the battery, and the alkaline at the zinc ſide, a deviation from analogy which I am not able to account for. The ſame inconſiſtency between the battery and the ſimple Galvanic chain is obſerved in their reſpective action on the organ of ſight, as the ſame pole of the battery and of the ſimple chain will produce oppoſite phenomena; that is to ſay, while one pole of the battery occaſions a ſtrong lightning, the ſame ſide of the ſimple Galvanic chain cauſes only a weak light, and vice verſa. The phenomena produced by the action of the battery are accordingly differently modified with reſpect to intenſity as well quality. The zinc ſide proves always more efficacious at the moment the chain is ſhut than the ſilver ſide; but when the chain is ſeparated, the ſhocks ariſe from the latter pole: On bringing the eye in contact with the zinc ſide, and the hand with the ſilver ſide, a reddiſh dim light will be ſeen the moment that the chain is ſhut, and ſome time after; but on ſeparating it, a ſhock is perceived at the ſilver ſide, together with the appearance of a ſtrong bluiſh light. When the ſilver ſide, however, is connected with the eye, a ſtrong bluiſh light will appear; but, on the ſeparation of the chain, the light becomes weak and reddiſh. A connection of the eye with the zinc ſide produces a ſhock, and the appearance of light and colour; whereas, whereas, on bringing the eye in contact with the filver fide, the laft phenomenon, without a fhock, will be perceived.

Although the appearance and fucceffion of the different phenomena of the Galvanic action are likely to vary according to the different degrees of excitability, they are all to be confidered as different modifications of the fame ftimulus, which for the cure of difeafes, ought to be properly adapted to the nature of each. I fhall finally add a few remarks on an opinion lately advanced by Doctors Ritter and Treviranus. Thefe gentlemen obferved, that on forming a Galvanic chain in the following order, *muscle*, *nerve*, *zinc*, *silver*, *nerve*, *muscle*; the nerve which is armed with zinc is almoft entirely deprived of excitability, while the other remains irritable; the excitability, however, may be refufcitated by changing the coatings of the nerves; whence they concluded, that Galvanifm poffeffes at the fame time the power of depreffing and exciting, according to the different conftruction of the chain. It appears, however, more probable, that the nerve armed with zinc has been rather over irritated, and as it were, obtunded by the power of the Galvanic ftimulus, which acting in a lefs degree on the nerve coated with filver, feems not to have been capable of extinguifhing the excitability in this nerve: For elfe, how can a ftimulus be thought to be at the fame time depreffing and exciting? an idea not at all adapted to our improved knowledge of organifation. I have befides frequently applied the Galvanifm of the battery to both ears without changing the conductors; but I never obferved that the power of hearing had been diminifhed at the zinc fide, or increafed at the filver fide, which ought to have taken place if the above fuppofition was probable; but, on the contrary, all the efficacy feems to be derived from the zinc pole. The fenfations occafioned by the application of Galvanifm have been obferved to return from time to time; for inftance, the lightning appears fome time after as evident as if the battery had been juft applied to the eye.

Sect. 5. *On the Diseases in which Galvanism may be successfully employed.* From what we have above ftated it appears, that Galvanifm always acts as the moft exciting power, under whatever circumftances and in whatever manner it may be applied; but though it chiefly affects the nervous fyftem, it alfo quickens the circulation of the blood, and eafily occafions palpitations of the heart in perfons that are naturally fubject to them. The circulation of the blood is particularly increafed in that part to which it is immediately conducted, where, at the fame time, it caufes confiderable congeftions of blood. The action of fimple and compound Galvanifm merely differs in degree; degree; the firft is to be applied to thofe parts only which are covered with a thin epidermis, and the latter requires the parts to be previoufly wetted. But whenever the fimple Galvanifm is applied to excoriated fpots, it obtains the additional property of acting as a *derivant*, by caufing the iffue of a great quantity of humours, which, however, properly originates in its exciting quality. In remarking the differences between Galvanifm and Electricity, I mentioned, that I had as yet been able only to act on fingle parts and on topical difeafes, as the whole body cannot be charged with Galvanifm in the fame manner as with Electricity, by being infulated. It is, however, in fome meafure practicable to act on the whole body by means of Galvanifm, when it is brought from top to toe within the chain of the battery. This experiment I have frequently tried on myfelf with a moderate battery, by touching with one conductor the top of the head, and with the other the fole of the foot, during which experiment I perceived a flight appearance of lightning, my head grew dull, and my eyes became red. We ought, however, to be cautious in making the experiment with a very ftrong battery, as it is to be expected the Galvanic ftream might pafs through the fkull to the brain and fpinal marrow, and produce the moft violent fymptoms, though fuch an experiment may be applicable in cafes of afphyxia. I gave in this manner five or fix fhocks to a rabbit, by applying one conductor of a battery of 150 ftrata to the forehead, which had been deprived of its hair and wetted, and the other to the os sacrum, by which the animal, however, was not killed but only moft violently convulfed. The poor creature feemed to be quite exhaufted after the experiment, it breathed with violent efforts, and became almoft fenfelefs. Several minutes after it crept away, and in the evening it began to eat again.

In general, difeafes from direct afthenia, originating in debility with increafed irritability, viz. in the generality of nervous and fpafmodic difeafes, the application of Galvanifm is not to be recommended, as, according to my experiments, it feems to do more harm than good in thefe cafes. The fpafmodic difeafe of a young man, which had the appearance of a tetanic affection, beginning with a fenfation in the feet, fimilar to the aura epileptica, got worfe by applying the Galvanic action to the feet; and likewife a nervous head-ach was not cured, notwithftanding the repeated application of Galvanifm. Perfons, whofe nerves are very irritable, are eafily affected with nervous fymptoms from the application of Galvanifm to any part of the body. As the general action of this ftimulus on the nerves confifts in producing contractions and convulfions, it is not deemed applicable applicable in ſpaſmodic diſeaſes, in which the nervous ſyſtem is naturally liable to the return of the convulſions without the acceſſion of any extraneous cauſe. It is even obſerved, that on galvaniſing ſingle parts of the body, the whole nervous ſyſtem ſeems to ſuffer, or the irritation to be propagated beyond the part to which it is properly conducted. Thus, on applying the zinc conductor to the knee, and the other to the back of the foot, a painful ſenſation extending up to the belly is perceived by many perſons. The diarrhœa, which Mr. Ritter experienced by remaining in contact with a battery of 100 ſtrata for above half an hour by means of his arms, is likewiſe to be accounted for from the irritation having diffuſed itſelf to the nerves, which were ſituated beyond the Galvanic chain. Galvaniſm, particularly when applied to the head, cauſes congeſtion of blood towards the head, dullneſs, tooth-ach, running at the noſe, a general drowſineſs, and inclination to ſleep: The patients on whom it has been properly employed enjoy a very good ſleep after it. But when thoſe ſymptoms increaſe, and the patient has reſtleſs nights, not much effect is to be expected from Galvaniſm. The diſeaſes in which I have tried the action of this ſtimulus were topical, originating from debility attended with deficient irritability, or paralytic complaints. I ſelected in the beginning ſuch caſes for my experiments, that had hitherto fruſtrated all medical aſſiſtance, and where no harm could be done; but after I had found it to have, in general, no bad conſequence on the conſtitution, I began to apply the Galvaniſm in other complaints. The diſeaſes in which I think the application of Galvaniſm is attended with effect, are the following.

1. *Paralysis of the Extremities.* If the application of Galvaniſm is intended to be of any uſe in theſe complaints, the cauſe of the diſeaſe ought to be only partial in the nerves themſelves, by their not having the proper degree of excitation. Paralyſis therefore ariſing from mechanical cauſes, exoſtoſes, contortions, and other leſions of the back bone, and preſſure on the brain, are not comprehended within the action of the ſtimulus. A paralyſis may have originated from apoplexy and a preſſure on the brain, but the cauſe muſt have ceaſed in order to admit the application of Galvaniſm. It is ſometimes extremely difficult to aſcertain, whether in hemiplegies the primitive cauſe continues to act in the brain, or whether the paralyſis only remained in conſequence of the debility of the nerves, ceſſante cauſa; but we may expect ſucceſs from Galvaniſm when life returns to any part of the deceaſed ſide; at all events the experiment will be quite harmleſs. In paralyſis ariſing from gout and chronic rheumatiſm, the application of the

Galvanic

Galvanic ſtimulus is properly indicated, particularly as it may be made to act alſo as a diſcutient and derivant, if it is conducted to bliſters. The cauſe of paralyſis from retrogade exanthemata muſt be firſt removed, before Galvaniſm is to be applied; but if the paralyſis ſhould remain, ſublata cauſa, it is time to order the uſe of Galvaniſm.

2. *Weakness of Sight and Amaurosis.* The application of Galvaniſm takes place in any degree of the diſeaſe, but not every ſpecies of amauroſis is qualified for the proper application of the Galvanic agent; according to my obſervations, we may only expect ſucceſs from it in that ſpecies which originates in a debility and paralyſis of the optic nerve, attended with deficient irritability, or in indirect debility. The diagnoſis of this particular ſtate is not ſo eaſy as may be imagined; but to trace the cauſe of this diſeaſe is frequently attended with great difficulties, though they are chiefly capable of throwing light on the true nature of the diſeaſe. Any cauſe which debilitates the whole nervous ſyſtem, and conſequently the optic nerve, or which immediately diminiſhes the excitability of the eye, viz. too ſtrong and laſting action of light on the eye, previous inflammations of the eyes, exceſſive uſe of the eyes, particularly in microſcopical obſervat[illegible]ns, action of morbid ſtimuli on the eye, gout, retrograde exanthemata. This ſtate is diſtinguiſhed by the following criteria. 1. When the ſight of the patient gets better, after he has taken a meal and drank ſome wine, than when he is faſting. 2. When he looks better after a walk. 3. When his ſight is improved in ſtrong ſun-light. 4. When the application of external ſtrong exciting remedies, as ſpirit ſal. ammoniac, has a favourable effect.

In that ſpecies of amauroſis, however, in which the debility and paralyſis of the optic nerve are united with increaſed excitability, I am induced to diſſuade the application of Galvaniſm. The patient ſees better in this caſe when the light is moderate; his eyes are very ſenſible and become painful by the leaſt effort; ſometimes he can hardly bear light at all, and the eyes water. Although Dr. Auguſtin likewiſe recommends the uſe of Galvaniſm in this caſe, adviſing to begin with a few ſtrata according to the Brunonian table, I have always obſerved by my experiments, that it is not only of no effect, but alſo is certainly prejudicial to the cure. It ſeems as if, in this caſe, another ſtimulus acted, producing together with debility a certain degree of excitement, which never admits the application of exciting remedies. I have at leaſt ſucceſsfully employed here gently and permanently ſtrengthening, opiate, and derivant remedies, *viz.* acids with opium, narcotic remedies, extractum

 hyoſciami, hyofciami, &c. Galvanifm is ftill lefs applicable in the amaurofis which arifes from the accumulation of blood, and from the diftenfion of the blood veffels near the optic nerve, or on the retina; a morbid ftate, which is frequently occafioned by fuppreffed menfes or hæmorrhoids. The amaurofis caufed by impurities of the primæ viæ, retrograde gout, rheumatifms, and exanthemata, are likewife not fit for the application of Galvanifm, as long as thofe caufes continue to act; but when amaurofis remains after their having ceafed, we may employ it with hopes of fuccefs.

In the amaurofis produced by violent commotions of the eye, I fhould likewife think it of ufe; but not much is to be expected in that fingular fpecies of amaurofis, which is occafioned by wounds of the eye-brows in the region where the frontal nerve iffues, and particularly by their fubfequent cicatrization.

3. *Difficulty of Hearing, and Deafness.* What has been ftated of amaurofis likewife takes place in the affections of hearing, particularly that we cannot expect advantages in all fpecies of deafnefs from the application of Galvanifm, but only in fuch, in which the proximate caufe confifts in a debility and paralyfis of the acouftic nerve, attended with deficient excitability. All caufes therefore, which by too vehement irritation have produced an indirect afthenia in the organs of hearing; too violent founds, previous inflammations of the meatus auditorius, typhus, &c. conftitute that ftate of deafnefs in which the application of Galvanifm may be ufed. The criteria of this ftate are the following. 1. The difeafe diminifhes or increafes according to the different degree of excitement of the patient, and according to the general ftate of health, of weather, and time of day. 2. The patient hears better when he is in good health, and in a proper degree of excitement, after a meal and in agreeable company, and when he is in good fpirits. 3. Dry and clear weather improves his hearing. 4. He hears better at night than in the morning; and when he has quietly and well refted, worfe than after a reftlefs night. 5. He hears more diftinctly when there is much and loud noife about him.

### EXPLANATION of TABLE II.

Fig. 1. A machine for faftening the conductors in the ears, by which the conductors can be turned in all directions. It confifts of a hoop of whale-bone, *a b*, which is faftened to a ftrap of leather, taffety or velvet, which can be buckled round the head, *c*, *c*, *c*. From each end of that hoop an arm of brafs with double joints, ending in a cafe, in which a glafs pillar, *g*, *h*, moves up and down; at the end of the glafs pillar is another cafe, *K*, through which paffes a glafs tube with the conductor.

Fig. 2. This machine applied to the head of a perfon, after being brought in contact with the pile.

Fig. 3. A conducting plate from which the chains proceed.

Fig. 4, 5, and 6. The fingle parts of the above machine.

*Observations*

## Figures and Tables

**Fig. 1. Fig. 2. Fig. 3. Fig. 4. Fig. 5. Fig. 6. f1:**